# Honey on brain health: A promising brain booster

**DOI:** 10.3389/fnagi.2022.1092596

**Published:** 2023-01-17

**Authors:** Nurul Ashykin Zamri, Nurhafizah Ghani, Che Aishah Nazariah Ismail, Rahimah Zakaria, Nazlahshaniza Shafin

**Affiliations:** ^1^Department of Physiology, School of Medical Sciences, Universiti Sains Malaysia, Kubang Kerian, Kelantan, Malaysia; ^2^School of Dental Sciences, Universiti Sains Malaysia, Kubang Kerian, Kelantan, Malaysia

**Keywords:** honey, antioxidants, brain health, memory booster, neuroprotective, anti-stress, antinociceptive, phenolic and flavonoid

## Abstract

Since ancient times, honey has been employed in many aspects of everyday life, the most popular of which is as a natural sweetener. Honey is used not only as a nutritional product but also in health as a supplement and in various applications, especially related to brain booster health. Brain health is the capacity to carry out all mental functions necessary for cognition, such as learning and judging, utilizing language, and recalling. This review presents the current trend of research on honey, particularly the interest in underlying mechanisms related to brain booster health. A total of 34 original articles addressing brain health from the consumption of honey were analyzed. We identified four main brain health benefits, which are memory booster, neuroprotective effect, anti-stress, and anti-nociceptive potentials with the proposed underlying mechanism. A lot of attention has been paid to the role that honey plays in brain health research, with the goal of examining the link between honey and brain health as well as the mechanism underlying it, the findings from this review may be potentially beneficial to develop new therapeutic roles for honey to help determine the best and most promising to benefit and boost overall brain health.

## 1. Introduction

Honey has been employed in many aspects of everyday life - the most popular of which is as a natural sweetener (Dan et al., 2018). The majority of honey’s health benefits have been anecdotal, based on observations and generalizations with no scientific evidence. However, there has been a renewed interest in exploring honey’s potential health benefits in the previous decade (Azman et al., 2021). Honey is used not only as a nutritional product but also in health as a supplement. Various applications of this product also can be found especially related to brain health.

Brain health can be defined as preserving optimal brain integrity and mental and cognitive function at a given age in the absence of overt brain diseases that affect normal brain function (Wang et al., 2020). For people to be independent, to engage in the things that mean most to them, and to be able to participate in life, a healthy brain is essential. The word “brain health” has no accepted definition as of yet (Alchalabi and Prather, 2021). According to the Centers for Disease Control and Prevention (CDC), brain health is the capacity to carry out all mental functions necessary for cognition, such as the capacity to learn and judge, utilize language, and recall (Day and Friedman, 2009). The phrase “brain health” focuses on maintaining the best possible mental health and improving cognitive performance to help each person to achieve his functioning in the areas of cognition, emotion, psychology, and behaviors. “An ounce of prevention is worth a pound of cure.” In order to keep a healthy brain, one must have normal growth, adaptability, promote healthy habits, adapt to stress and adversity, and develop resilience to deal with the shifting demands of daily life (World Health Organization, 2022).

Since the beginning of time, honey has been utilized as a natural quick food. It is a viscous, sticky fluid that bees and other insects make from nectar gathered from flowers and use as nourishment. The type of bees and insects that gather the nectar, as well as the source flower from which it is obtained, are used to categorize honey (Yahaya et al., 2020). Honey is regarded as a healthy food with equal appeal for men and women of all ages (Bell, 2007). Honey does not require chilling, does not go rancid, and may be kept unopened at room temperature in a dry spot. Honey’s water activity ranges from 0.56 to 0.62, and its pH level is about 3.9 with a specific gravity of 1.34 (Hassapidou et al., 2006; Moniruzzaman et al., 2014). Honey contains 200 substances, and the physiochemical composition of different types of honey is summarized and shown in Table 1.

**Table 1:** Summary of the physiochemical properties of honey per 100 g (Terzo et al., 2020).

Physiochemical properties	Honey	Physiochemical properties	Honey
Physical appearance	Light to dark brown	Calorie	304 cal
Specific gravity	1.34	Lipids	0.02 g
pH	± 3.9	Organic acid	
Water activity	0.56 to 0.62	Free acids	0.43 g
Water content	17.1 g	Lactone	0.14 g
Total sugar	79.7 g	Total minerals	
Monosaccharides		Potassium, K	52.0 mg
Fructose	38.2 g	Sodium, Na	10.0 mg
Glucose	31.3 g	Calcium, Ca	6.0 mg
Disaccharides		Iodine, I	10.0 mg
Sucrose	0.7 g	Phosphorus, P	4.0 mg
Others	5.0 g	Magnesium, Mg	2.0 mg
Trisaccharides		Fluoride, F	1.07 mg
Melezitose	<0.1 g	Manganese, Mn	0.5 mg
Erlose	0.8 g	Iron, Fe	0.42 mg
Others	0.5 g	Copper, Cu	0.3 mg
Undetermined oligosaccharides	3.1 g	Zinc, Zn	0.22 mg
Amino acids	0.3 g	Selenium, Se	0.02 mg

**Figure 1:**
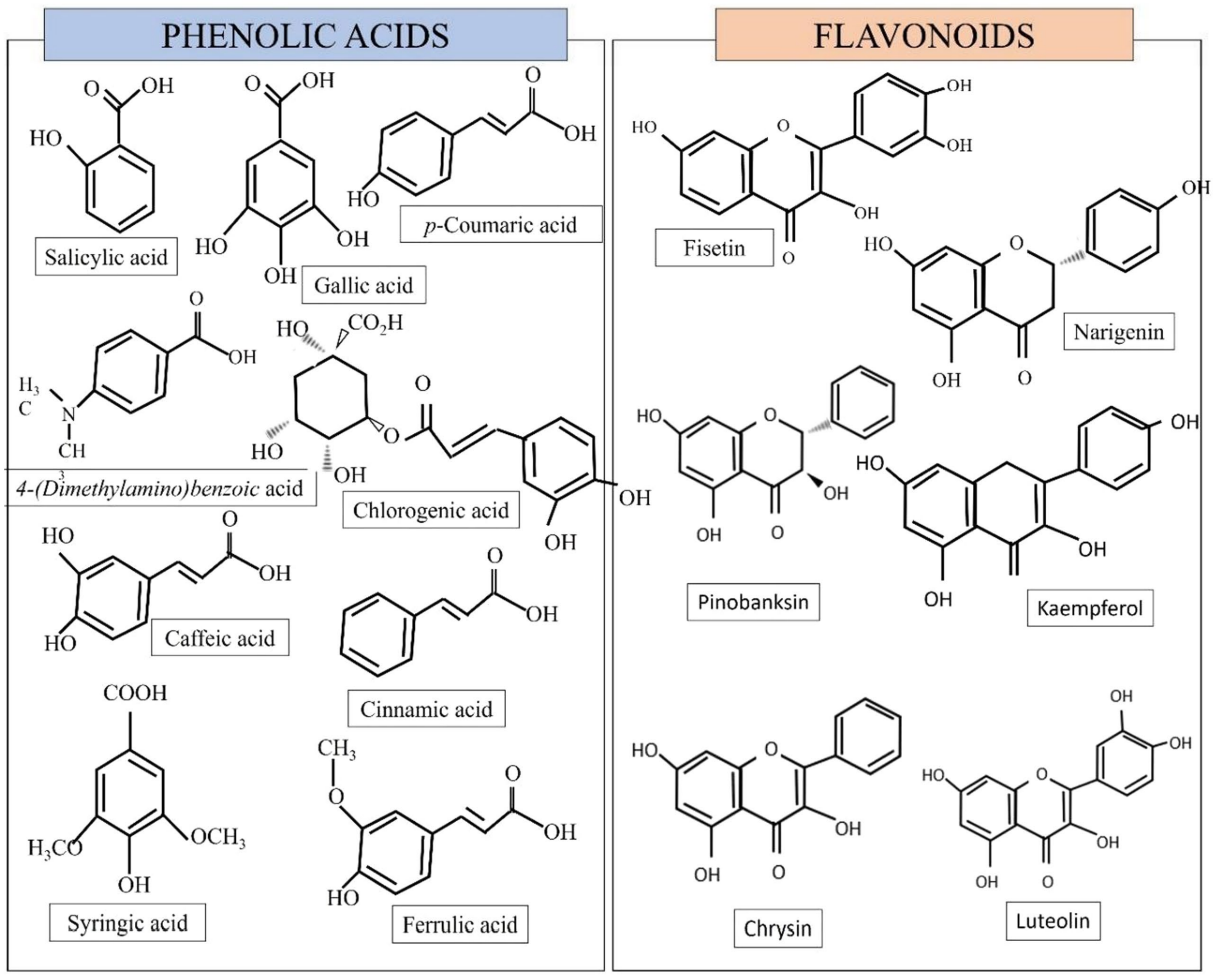
Some of the phenolic and flavonoid compounds found in honey (Biluca et al., 2017; Azman et al., 2021).

Honey’s sugar composition is mostly constituted of monosaccharides such as fructose and glucose, as well as disaccharides such as sucrose and maltose (Chua and Adnan, 2014). The main bioactive molecules present in honey are represented by polyphenols. Polyphenols can be divided into flavonoids and phenolic acids (Miguel et al., 2017). Honey contains a variety of phenolic acids (gallic, coumaric, syringic, caffeic, cinnamic, benzoic, chlorogenic, salicylic, and ferulic acid) as well as flavonoids (catechin, quercetin, kaempferol, luteolin, hesperetin, apigenin, 3,7,40-trihydroxyflavone, naringenin, chrysin, fisetin, vitexin, isoorientin, xanthohumol pinobanksin-3-o-propionate and pinobanksin-3-o-butyratengenin) (Khalil et al., 2011; Nurul et al., 2013; Chew et al., 2018). Some of the polyphenol contents are illustrated in Figure 1.

Some honey is kept in clusters of little resin pots near its nest. Meanwhile, most of the honey produced by naturally occurring bees is kept in hexagonal-shaped combs (Kek et al., 2014). Some honey is more diluted than other varieties of honey, and some have a particular flavor (sour-like flavor) as well as scent (Biluca et al., 2014).

Honey has previously been used to fight aging, boost libido and the immune system, destroy germs, heal bronchial phlegm, and relieve sore throat, cough, and cold symptoms. Honey has been shown to have several pharmacological effects, including anti-inflammatory, antioxidant, anti-aging, and antibacterial activities. Because honey has excellent antioxidant properties, it might be one of the natural chemo-preventive agents (Saiful Yazan et al., 2016).

To better understand the relationship between honey, brain health, and their mechanisms, emphasis has been given to the role that honey plays in brain health studies. Thus, this article highlights the current literature on honey, specifically emphasis on its brain health effects. The possible underlying mechanisms of its brain health effects are also discussed.

## 2. Method

For this systematic search, we developed a search strategy to identify relevant works of literature. This search strategy was tailored to three databases: PubMed, Scopus, and ScienceDirect restricted to English articles. The following keywords were used individually and in combination as inclusion criteria for articles to be considered for this review (“Honey” AND “Brain” AND (memory booster OR stress OR antinociceptive OR antidepressant OR anxiety-like OR antistress OR cognitive OR neuroprotective OR neurodegeneration OR analgesic OR inflammation OR neuroinflammation’)).

**Figure 2:**
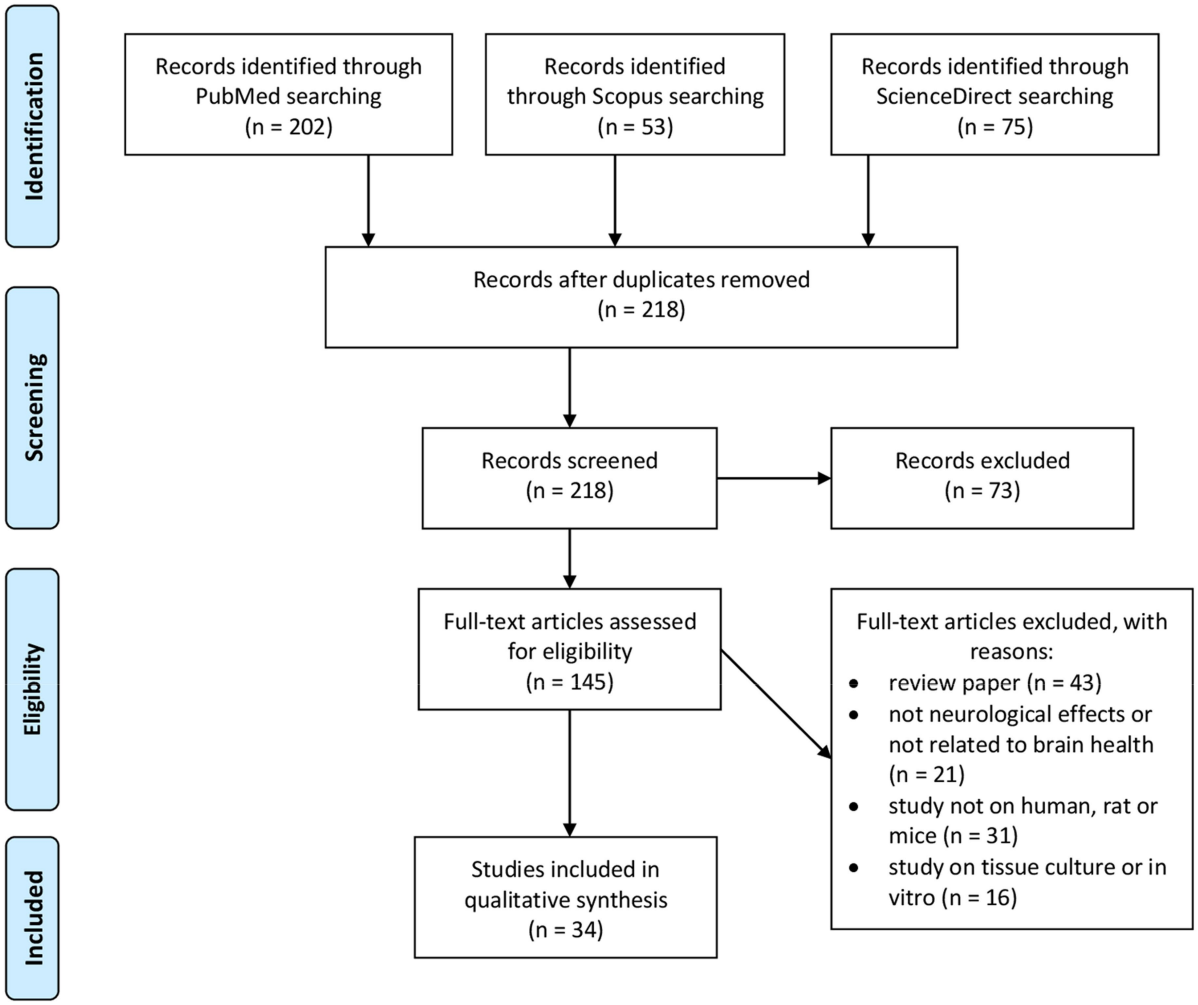
Flow diagram of PRISMA statement for inclusion and exclusion criteria at every stage.

The present review covers studies based on honey consumption to investigate its effects on brain health. Initial searches yielded 330 results. The abstracts of these papers were reviewed to confirm applicability. After considering additional exclusion criteria 34 papers remained as shown in Figure 2.

The selection criteria were based on the PRISMA statement (Moher et al., 2009). The searches were mainly focused on the mapping of existing literature on honey in the article title, abstract or keyword related to honey, brain health, neurological effects and the nervous system. The review papers, theworks of literatures that did not address the neurological effects or brain health, and the study used other than human or rat or mice as well as cell culture are excluded. The study included only original articles published in journals. All duplications were thoroughly checked *via* Endnote. Abstracts of the articles were checked, and purification of the articles was performed to ensure the quality and relevance of the academic literature which were going to be included in the review process. A careful evaluation of each research paper was carried out at a later stage.

## 3. Results and discussion

There were 34 articles examined, all of which were focused on human and animal studies. The memory enhancer, neuroprotective impact, anti-stress, and anti-nociceptive potential benefits of honey have been divided into four main groups. Additionally, it critically discusses the mechanism and suggests the benefits, particularly concerning neurocognitive and brain function.

There are numerous studies looking at the effects of honey on medicinal properties, however, there is a scarcity of topic on the effect of honey on brain health, especially in humans as tabulated in Table 2. Among the first to study the effect of honey and showed a promising brain booster is a study on the effect of honey on cognition by Al-Himyari (2009).

**Table 2:** Memory booster effects of honey supplementation on human and animal models.

**Authors (Year)**	**Type of source**	**Purpose**	**Mechanism**
Othman et al. (2011)	Human study (postmenopausal women)	To evaluate the verbal learning and memory performance of postmenopausal women who received honey in comparison with women receiving estrogen plus progestin therapy and untreated controls.	Honey is a phytoestrogen, and it may have neuroprotective benefits by upregulating the production of BDNF which acts as an antioxidant
Al-Rahbi et al. (2014b)	Animal study (rat)	To examine the effects of the honey supplement on hippocampal morphology and memory performance in-stressed ovariectomised rats exposed to social instability stress.	Honey treatments increased hippocampus CA2, CA3, and dentate gyrus (DG) area neuronal proliferation and boosted short- and long-term memory.
Azman et al. (2015)	Animal study (rat)	To investigate the potential protective effects of honey supplementation on memory performance in aged rats exposed to noise stress.	Improved neuronal proliferation in the mPFC and hippocampus, reduced oxidative stress levels, and elevated BDNF level.
Arshad et al. (2020)	Animal study (rat)	To investigate the effects of honey on the brain of Metabolic Syndrome-induced rats.	Honey can normalize blood sugar and lower serum triglyceride and LDL levels, and behavioral tests support its effects on anxiety and memory
Yaacob et al. (2020)	Animal study (rat)	To investigate the cognitive-enhancing effects of honey and its methanolic fraction in comparison to the clinically approved NMDA receptor antagonist (memantine) using the LPS rat model.	Both honey and its methanolic fraction improved spatial and recognition memory of LPS rats and benefits for neuroinflammatory neurodegenerative disorders
Abd Aziz et al. (2020)	Animal study (rat)	To investigate the alterations in memory and hippocampus morphology and levels of MDA and NMDA receptors in the hippocampus of adult rats after prenatal stress could be prevented by the administration of honey.	Increased number of hippocampus neurons (neurogenesis) after honey injection
Yahaya et al. (2020)	Human study (schizophrenia patients)	To explore the effect of honey on cognitive domains, especially as it pertained to the verbal memory of schizophrenia patients.	Increased memory function affected by choline and Ach levels in addition to acting as an antioxidant
Al-Himyari (2009)	Human study (Mild cognitive impaired patients)	To investigate the role of honey in slowing the progression of dementia and if it has any anti-amyloid properties.	It improves the cholinergic system and blood flow in the brain and has antioxidant effects.

### 3.1. Memory booster

Honey was found to exhibit memory booster effects in both experimental rats and humans as tabulated in Table 2. In prenatal stress rats, it might prevent memory impairment, and alterations in MDA and NMDA receptor levels compared to stress and control groups. The findings suggested that honey consumption has benefits on prenatally stressed rat pups where it significantly lowered the level of MDA and NMDA receptors with higher neuronal numbers compared to the stress group (Abd Aziz et al., 2020). In a mouse model of Down’s syndrome, luteolin, one of the flavonoids in honey, has been demonstrated to promote neurogenesis in the hippocampus. An increase in neurogenesis was linked to better learning and memory performance (Yu et al., 2019). Increased neurogenesis in the rat progeny, which is linked to better recognition memory, is suggested by the increased hippocampus neurons after honey injection in pregnant females.

Another experimental rat study summarized that honey consumption improved neuronal proliferation in the medial prefrontal cortex (mPFC) and hippocampus by reducing oxidative stress levels, elevating brain-derived neurotrophic factor (BDNF) levels, and reducing acetylcholinesterase activity (Azman et al., 2015). Honey is a phytoestrogen, and it may have neuroprotective benefits by upregulating the production of BDNF which acts as an antioxidant (Al-Rahbi et al., 2014a). Honey supplementation was able to decrease oxidative stress levels and acetylcholinesterase activity to increase BDNF concentration and neuronal proliferation in the medial prefrontal cortex and hippocampus and attenuate memory deficits (Azman et al., 2015).

Another recent clinical trial on honey involved schizophrenia patients, and it found that 8 weeks of honey intake enhanced overall learning across domains in short-term memory yet not in long-term memory (Yahaya et al., 2020). Honey has the significant total phenolic content and is high in flavonoids. Therefore, honey could have an impact on choline and Ach levels in addition to improve antioxidant ability which critical for memory formation (Khalil et al., 2011).

Sixteen weeks of honey intake in postmenopausal women led to improvements in short-term memory and oxidative stress levels equivalent to those attained by patients undergoing estrogen with progestin treatment. The progress in total learning and immediate memory enhancement is likely best explained by honey consumption and not attributed to estrogen alone.

Furthermore, when compared to stressed ovariectomized rats that treated, honey treatments increased hippocampus CA2, CA3, and dentate gyrus (DG) area neuronal proliferation and boosted short- and long-term memory. When treated with honey, the cortisone levels in stressed ovariectomized rats may reduce from potential HPA axis interaction (Al-Rahbi et al., 2014a).

In addition, from the metabolic syndrome rats’ elucidation, honey can normalize blood sugar and lower serum triglyceride and LDL levels, and behavioral tests support its effects on anxiety and memory (Arshad et al., 2020). Yaacob et al. (2020) found that the lipopolysaccharide (LPS) rat model greatly reduces spatial and recognition memory through honey consumption. Honey and its methanolic fraction has have potential therapeutic and preventative benefits for neuroinflammatory and neurodegenerative disorders (Yaacob et al., 2020).

In the earliest study using honey, a total of 2,893 individuals 65 years of age and older were included in this research between November 2003 and November 2008. After the consumption of honey for 5 years, only 489 subjects developed dementia. This research by Al-Himyari (2009) set out that honey has memory booster effects to treat dementia and cognitive deterioration. It improves the cholinergic system and blood flow in the brain and has antioxidant effects. Additional research is required to determine whether honey slows the course of dementia and whether it possesses any anti-amyloid effects (Al-Himyari, 2009).

### 3.2. Neuroprotective effects

A well-known pneumo-toxicant and dopaminergic neurotoxic, paraquat (PQ) damages cells as a result from oxidative stress, which causes toxicity. Honey therapy reduced the toxic effects seen in the lungs and midbrain. This indicates honey may act as an antioxidant defence mechanism to protect dopaminergic neurons from oxidative stress-related damage (Tang et al., 2017).

One study investigated the extent of the neuroprotective effect conferred by honey, as an antioxidant agent, in the cerebral cortex of rats against KA-induced oxidative stress and neurodegeneration in an animal model of KA-induced excitotoxicity (Mohd Sairazi et al., 2017). The neurotoxin kainic acid (KA), which is obtained from the red alga Digenea simplex, is frequently used to cause seizures and used in a study of relationship between excitotoxicity and neurodegeneration. The cerebral cortex’s increased levels of thiobarbituric acid reactive chemicals and decreased total antioxidant status level, both of which were amplified by KA, were dramatically mitigated by honey administration (Mohd Sairazi et al., 2017).

By over-activating glutamate receptors, KA has enhanced the generation of ROS, which are oxidative stress mediators. According to some theories, oxidative stress may be a key factor in the process behind excitotoxicity and neurodegeneration in many brain areas. When cells are under oxidative stress, ROS are produced, which oxidize DNA, protein, and membrane lipids (Bruce and Baudry, 1995; Gluck et al., 2000).

Learning and memory are two cognitive processes controlled by the CNS. Results from earlier research indicate that the hippocampus, which is essential in spatial learning and memory, is susceptible to hypoxia (Maiti et al., 2008). Memory impairment has been linked to the hippocampus being affected by hypoxia exposure (Malle et al., 2013; Qaid et al., 2017). This study hypothesized that honey pretreatment has protective benefits against memory losses brought on by hypoxia, presumably due to its antioxidant components (Qaid et al., 2020a).

Epileptic seizures were brought on by the administration of KA. In the rat cerebral cortex, cerebellum, and brainstem, KA increased the levels of tumor necrosis factor-alpha (TNF-α), interleukin 1 beta (IL-1β), glial fibrillary acidic protein (GFAP), allograft inflammatory factor 1 (AIF-1), and cyclooxygenase-2 (COX-2), as well as the activation of caspase-3. These factors affect how neurodegeneration manifests itself (Oprica et al., 2003; Kwon et al., 2010).

Caspase-3 activity following status epilepticus produced by kainic acid (KA) was decreased by honey (Mohd Sairazi et al., 2018). Pretreatment with honey simultaneously lowered proinflammatory cytokine production (reduced the elevation of TNF- and IL-1 levels) and inhibited microglial and astrocyte activation (reduced the elevation of GFAP and AIF-1 levels). This data thus indicates that in this KA-induced excitotoxicity paradigm, honey exerts its neuroprotective impact *via* its anti-inflammatory mechanism (Mohd Sairazi et al., 2018).

Information transport and storage are tightly tied to the connections of neural networks with the hippocampus and prefrontal cortex (PFC) at their centres (Schwindel and McNaughton, 2011). Interactions between the prefrontal and hippocampal networks are disrupted by hypoxia-ischemic damage, which may have an impact on cognition and behavior (Brockmann et al., 2013). In male rats subjected to normobaric hypoxia, honey improves brain cholinergic indicators, protecting against hypoxia-induced medial prefrontal cortex (mPFC) neuronal damage (Qaid et al., 2021).

According to our research, normobaric hypoxia impairs the antioxidant system’s effectiveness, which causes oxidative damage. It has been shown that pretreatment with honey increases brain resistance to hypoxia (Qaid et al., 2020b). These findings imply that antioxidant enzymes might be upregulated in rats in both normoxic and hypoxic environments by honey. Honey antioxidant characteristics, include flavonoids (catechin, kaempferol, naringenin, luteolin, and apigenin) and phenolic acids (gallic, syringic, benzoic, trans-cinnamic, p-coumaric, and caffeic acids) are likely contributing factors to the beneficial effect on rats’ brains (Qaid et al., 2020b).

In comparison to Nigella sativa oil, honey was shown to have more substantial impacts on neuronal soma size (Mohd Yusoff et al., 2018)because the neuronal soma integrates incoming information. It is possible to interpret the bigger somas as having superior cellular preservation and memory capacity. It may indicate improved memory and learning capacity. To support a bigger neural dendritic tree, more synaptic connections, and greater neuronal activity, a larger soma may contain larger cellular and metabolic systems. Thus, they equate to greater memory capacity (Freas et al., 2013).

One of the most significant flavonoids contents is pinocembrin, which was identified from propolis and honey. It has antioxidant, antibacterial, and anti-inflammatory properties (Estevinho et al., 2008; Feng et al., 2012). Pinocembrin reduced oxidative stress, inflammatory, and apoptotic indicators as well as glutamate and lactate dehydrogenase activity to lessen the harm caused by cerebral ischemia–reperfusion. The most important conclusion was that pinocembrin normalized the enlarged infarct size brought on by cerebral ischemia–reperfusion (Saad et al., 2015).

Chronic aluminium (Al) exposure can hasten the onset of many neurological and neurodegenerative conditions. Al is a metal that encourages oxidative damage, which results in neuronal death in various parts of the brain and deficiencies in behavior, cognition, and memory (McLachlan et al., 2018; Exley and Mold, 2019). Chrysin, in particular, restored the acetylcholinesterase and butyrylcholinesterase activity in the hippocampus and lessened the cognitive impairment brought on by AlCl_3_. The oxidative damage to the cerebral cortex and hippocampus’s lipid peroxidation, protein carbonylation, catalase, and superoxide dismutase was mitigated by the chrysin. Last but not least, chrysin treatment also reduced the frequency of necrotic cells in the same brain areas (Campos et al., 2022).

In addition, the study by Goes et al. (2018) also demonstrated the chrysin administration for neuroprotective effect in neuroinflammation, neurotrophic factors and neuronal recovery factors a recognized model of Parkinson’s disease, in the striatum of mice. The chrysin administration has proven that it was able to increase levels of TNF-α, IFN**-γ**, IL-1β, IL-2, IL-6, and nuclear factor kappa B (NF-κB) and decreased the IL-10 levels, total reactive antioxidant potential and total antioxidant reactivity in the striatum, as well as, modified the calcium-binding protein B (S100B), BDNF, nerve growth factor and glial cell line-derived neurotrophic factor levels (Goes et al., 2018).

Moreover, there is proof that metabolic syndrome increases the risk of neurodegenerative diseases like Alzheimer’s disease which can lower cognitive function (Van Dyken and Lacoste, 2018). The high antioxidant content in honey such as caffeic acid significantly reversed the hyperglycemic and hypertension in metabolic syndrome rats. Besides that, level of brain TNF-α levels have significantly reduced as well as increased the brain BDNF levels (Kadar et al., 2022). According to this result, honey and caffeic acid may be able to reduce the effects of high-carbohydrate high-fructose-induced metabolic syndrome and have neuroprotective properties (Kadar et al., 2022).

Finding by Muhammad et al. (2014) demonstrated that injection of sodium arsenite induced lipid peroxidation with associated detrimental effects on enzymatic antioxidants which alleviated in the presence of honey administration. Sodium arsenite is known as clastogen which is able to cause chromosome breakage which can eventually elevate lipid peroxidation (Aliyu and Odunola, 2012; Bhattacharya and Haldar, 2012). The honey administration has significantly reduced the MDA levels, superoxide dismutase (SOD), catalase, and glutathione peroxidase activities (Muhammad et al., 2014).

In addition, the study by Abdulmajeed et al. (2015) summarized that in lead-exposed rats, honey boosted mobility, encouraged exploration, and reduced anxiety. Also, administering honey boosts antioxidant activities as demonstrated by a rise in brain SOD, GST, and GSH activities in comparison to those who had been exposed to lead (Abdulmajeed et al., 2015). We may conclude that honey protects against lead-induced cognitive loss, likely through boosting antioxidant activities.

Likewise, the lipopolysaccharides-induced neuroinflammation rats that consumed honey exhibited that honey consumption can significantly ameliorate the lipopolysaccharides-induced neuroimpairment as well as reduced the TNF- α and IL6 level. Besides that, it also can significantly reduce the malondialdehyde and nitrite levels in rat brains and reversed the depletion of reduced glutathione levels. Acetylcholinesterase activity in lipopolysaccharides-induced neuroinflammation rat brains was reduced by honey. Compared to the LPS-only group, the hippocampus, prefrontal cortex, and striatum revealed the restoration of neuronal structure and Nissl body distribution by cresyl violet staining (Adeniyi et al., 2022).

In addition, a total 60 patients with the major neurocognitive disorder (MCD) from Iran were included to receive honey consumption. Both the Mini-Mental State Examination (MMSE) results and the GDS results during the intervention were statistically significant in the intervention group (*p* = 0.001, *p* = 0.004, respectively). In the brain tissue, honey reduces lipid peroxidation and simultaneously boosts the activities of glutathione reductase and superoxide dismutase based on Table 3 (Akouchekian et al., 2018).

**Table 3:** Neuroprotective effects of honey supplementation on animal models.

**Authors (Year)**	**Type of source**	**Purpose**	**Mechanism**
Tang et al. (2017)	Animal study (rat)	To investigate the protective effects of honey against PQ-induced toxicity in the midbrain and lungs of rats.	Honey may act as an antioxidant defence mechanism to protect dopaminergic neurons from oxidative stress-related damage
Mohd Sairazi et al. (2017)	Animal study (rat)	To investigate the extent of the neuroprotective effect conferred by Malaysian honey, an antioxidant agent, in the cerebral cortex of rats against KA-induced oxidative stress and neurodegeneration in an animal model of KA-induced excitotoxicity.	The cerebral cortex’s decreased levels of thiobarbituric acid reactive chemicals and increased total antioxidant status level upon honey consumption.
Qaid et al. (2021)	Animal study (rat)	to investigate the role of honey on medial prefrontal cortical neuronal morphology and cholinergic markers such as acetylcholine (ACh) and acetylcholinesterase (AChE) following exposure to normobaric hypoxia in rats.	Honey improves brain cholinergic indicators, protecting against hypoxia-induced mPFC neuronal damage
Qaid et al. (2020b)	Animal study (rat)	To investigate the responses of antioxidant defences to normobaric hypoxia and the effects of honey on the brain oxidant/ antioxidant status of adult male rats.	Honey increases brain resistance to hypoxia and antioxidant enzymes might be upregulated in rats in both normoxic and hypoxic environments
Mohd Yusoff et al. (2018)	Animal study (rat)	To focus on its morphometric effect on cornuammonis1 (CA1) pyramidal neurons of the hippocampus	Honey was shown to have more substantial impacts on neuronal soma size for a bigger neural dendritic tree, more synaptic connections, and greater neuronal activity
Akouchekian et al. (2018)	Human study (Major neurocognitive disorder patients)	To investigate the efficacy of the herbal combination of sedge, saffron, and Astragalus honey on cognitive and depression score of patients with the major neurocognitive disorder (MCD)	In the brain tissue, honey reduces lipid peroxidation and simultaneously boosts the activities of glutathione reductase and superoxide dismutase
Muhammad et al. (2014)	Animal study (rat)	To investigate the effect of Acacia honey from north-west Nigeria on sodium arsenite-induced oxidative damage and clastogenicity in male Wistar rats	The honey from Nigeria may possess high hydrogen peroxide scavenging activity to mitigate oxidative stress and clastogenicity
Goes et al. (2018)	Animal study (mice)	To investigate the possible involvement of inflammatory cytokines, neurotrophic factors and neuronal recovery in the effect of chrysin in 6-hydroxy dopamine (6-OHDA), a well-established model of Parkinson’s disease, in the striatum of mice.	The neuroprotective effect of chrysin from honey in the treatment of Parkinson’s disease and, indicated the mechanism involved through the inflammatory cytokines, neurotrophic factors and recovery of dopaminergic neurons in the striatum.
Saad et al. (2015)	Animal study (rat)	To determine the possible mechanisms of neuroprotection elicited by pinocembrin with specific emphasis on chronic prophylactic use before the induction of global cerebral ischemia–reperfusion	Pinocembrin reduced oxidative stress, inflammatory, and apoptotic indicators as well as glutamate and lactate dehydrogenase activity to lessen the harm caused by cerebral ischemia–reperfusion
Campos et al. (2022)	Animal study (rat)	To evaluate the antioxidant and neuroprotective effects of chrysin against the neurotoxicity elicited by aluminium chloride (AlCl_3_).	In particular, chrysin reduced the cognitive impairment induced by AlCl_3_ as well as normalized the acetylcholinesterase and butyrylcholinesterase activities in the hippocampus. The chrysin counteracted the oxidative damage, in terms of lipid peroxidation, protein carbonylation, catalase, and superoxide dismutase impairment, in the brain cortex and hippocampus. Lastly, necrotic cells frequency in the same brain regions was also decreased by chrysin administration
Nassar et al. (2020)	Animal study (rat)	to examine the protective role of bee products: a mixture of honey, propolis, palm pollen, and royal jelly (HPPJ) against Sumithion-induced toxicity.	The protective role of bee products: a mixture of honey, propolis, palm pollen, and royal jelly (HPPJ) counteracted the hematological, renal, and hepatic toxicity of sumithion exposure.
Kadar et al. (2022)	Animal study (rat)	To investigate the effects of honey supplementation and to compare it with a pure form of antioxidant, caffeic acid (CA), on MetS parameters and inflammatory markers in the brains of MetS-induced rats.	Honey consumption significantly reduced rain TNF-α levels and increased brain BDNF levels.
Abdulmajeed et al. (2015)	Animal study (rat)	To investigate the possible neuroprotective effects of honey against lead (Pb)-induced neurotoxicity.	Honey has neuroprotective effects against lead-induced cognitive deficit probably by enhancing antioxidant activities.
Adeniyi et al. (2022)	Animal study (rat)	To evaluate the ameliorative potential of honey in lipopolysaccharides-induced neuroinflammation.	Honey reduces TNF- α, IL6, nitrite and malondialdehyde levels and increases glutathione. Then, the positive restoration of neuronal structure and Nissl body distribution in the hippocampus, prefrontal cortex, and striatum.

### 3.3. Anti-stress

According to this study, noise stress was demonstrated to have a significant impact on cognitive performance, somehow the effects were mitigated by the honey supplement as tabulated on Table 4. These data imply that subchronic noise stress promotes depressive-like behavior and decreases cognitive processes. Honey intake was found to mitigate these effects (Azman et al., 2015).

**Table 4:** Anti-stress or anti-depressive effects of honey supplementation on animal models.

**Authors (Year)**	**Type of source**	**Purpose**	**Mechanism**
Azman et al. (2015)	Animal study (rat)	To examine the effects of honey supplements administered to prevent or attenuate the occurrence of stress-related behaviors in male rats subjected to noise stress.	Honey intake is able to mitigate the depressive-like behavior and cognitive processes linked to the HPA axis.
Al-Rahbi et al. (2014a)	Animal study (rat)	To evaluate the effects of honey, a phytoestrogen, and 17 β -estradiol (E2) on depressive-like behavior, stress hormones, and BDNF concentration in stressed ovariectomised (OVX) rats.	Honey consumption restores antidepressive-like effects in stressed OVX mice linked to the HPA axis and increases BDNF levels
Asari et al. (2019)	Animal study (rat)	To investigate the effects of honey, DHA-rich fish oil, and their combination on the concentrations of selected pro-inflammatory cytokines in rat brains following exposure to chronic stress.	Honey can reduce pro-inflammatory cytokine and corticosterone levels in rats’ brains under prolonged stress circumstances
Arabmoazzen et al. (2015)	Animal study (rat)	To investigate the effect of oral administration of honey on serum glucose, lipids, stress oxidative markers, and morphology of Langerhans islets in noise-induced hyperglycemic rats	Honey treatment significantly ameliorated the increased malondialdehyde (MDA) content and reduced the activity of superoxide dismutase (SOD) in the brain.
Filho et al. (2016)	Animal study (rat)	To investigate the action of chrysin treatment (5 or 20 mg/kg) for 14 days in the depressant-like behavior and in the hippocampal dysfunction induced by olfactory bulbectomy, an animal model of agitated depression.	Chrysin therapy prevented all the alterations in the hippocampus
Badrasawi et al. (2013)	Human study (depressed elderly patients)	To determine the effect of Talbinah on mood and depression among institutionalized elderly people in Seremban.	Honey consumption is able to improve mood by composition of amino acid (trp:BCAA and trp:LNCAA) and mineral (zinc and magnesium).

Both humans and rats rely heavily on the hippocampus for spatial memory. The hippocampus is also linked to the hypothalamic–pituitary–adrenal (HPA) axis and is sensitive to stress (McGirr, 2010). The HPA axis is hyperactivated by chronic stress, leading to the production of adrenocorticotropic hormone (ACTH) and corticosterone. This can cause structural alterations, cell shrinkage, and neuronal death in the hippocampus (McEwen, 2006). The dysregulation of the HPA axis has also been related to the maintenance and initiation of depression (Checkley, 1996).

These summarized that honey consumption has been shown to significantly reduce ACTH and corticosterone levels, as well as depressive-like behavior in rats. It is also suggested that honey improves cognition and reduces depressive symptoms in stress-induced rats due to its antioxidant capability, which is attributable to flavonoid levels. Honey contains a high flavonoid concentration ranging from 60 to 460 g/100 g of honey (Bogdanov et al., 2008). Honey consumption can significantly lower the ACTH and corticosterone blood levels in stressed ovariectomized rats compared to other experimental groups. Therefore, honey has the potential potetial to become anti-stress effects by restoring the hypothalamic–pituitary–adrenal axisd and increasing BDNF levels (Al-Rahbi et al., 2014a,b).

The phenylalanine in honey may upregulatebrain-derived neurotrophic factor (BDNF) in honey-treated rats and reduce the depressive-like behavior (Mustafa et al., 2019). BDNF is one of the numerous endogenous proteins that play an important role in the pathogenesis of several brain illnesses, including Huntington’s disease (Canals, 2004) and Alzheimer’s disease (Zhang et al., 2006). Reduced BDNF levels have also been linked to a variety of mental illnesses, including depression (Shimizu et al., 2003). In experimental animals, this causes to hippocampal shrinkage and neuronal loss (McEwen, 1999), which is supported by lower hippocampus volume in depressed people (Manji et al., 2001).

In another experimental rat, TNF-α, IL6, and IFN-γ concentrations in brain homogenates from the DHA-rich fish oil (DHA), honey, and honey + DHA-treated groups were considerably lower than in the control and stress-only-exposed groups (*p* < 0.05) (Asari et al., 2019). TNF-α, IL6, and IFN-γ concentrations are increased in most inflammatory conditions and are recognized as therapeutic targets (Scheller et al., 2011). TNF-α is a powerful immune system activator and involved in various basic physiological functions such as cell survival, gene expression, and synaptic integrity. TNF-α is generated by activated microglia, which can trigger TNF- production resulting in dysregulation of the inflammatory response in the central nervous system (Frankola et al., 2011).

Likewise, IL6 stimulates immunological responses. Moreover, IL6 is a powerful inducer of the acute phase response and modulates the transition from acute to chronic inflammation (Erić and Konjevic, 2014). The balance of inflammatory and anti-inflammatory cytokines is critical for optimal health. Any imbalance between these two cytokine types might cause dysregulation of the cytokine network. As a result, various disorders have emerged (Wojdasiewicz et al., 2014). This disclosed that the consumption of DHA-rich fish oil and honey can reduce pro-inflammatory cytokine levels in rats’ brains under prolonged stress circumstances. This research also shows that honey can reduce stress-induced increases in corticosterone levels, indicating that honey has anti-stress effects.

In another study using honey, the researcher selected 30 depressed senior people (21 men and 9 women) as a sample for 3 weeks period to use Talbinah with honey (Wurtman et al., 2003). Talbinah is a barley syrup that is prepared with milk and honey for sweetness. The Prophet Mohammad (SAW) advocated Talbinah in his well-known Hadith on the herb for its ability to calm hearts and lessen grief during terrible occurrences. In the intervention group, there was a statistically significant decline in the scores for depression, stress, and mood disturbances (*p* 0.05 for all parameters), according to a Wilcoxon nonparametric test (Badrasawi et al., 2013). A high carbohydrate diet from Talbinah (22.9 g per 100 kcal) can affect stress and mood. The amino acid composition in Talbinah which are trp: BCAA and trp: LNCAA are linked to the level of serotonin in the brain. The amount of tryptophan the brain could access may have risen due to the trp: BCAA ratio (Wurtman et al., 2003).

Nowadays, noise pollution is getting worse, especially in developed nations. Noise prevalence is linked to a number of human diseases and is account for the rising morbidity brought on by modern lifestyles (Mahmood et al., 2004). The study by Arabmoazzen et al. (2015) demonstrated noise for hyperglycemic induction. Treatment with honey dramatically lowered the brain’s elevated levels of malondialdehyde (MDA) and superoxide dismutase (SOD) activity. The quantity and granularity of beta cells were decreased in the hyperglycemic group’s Langerhans islets on histology; honey therapy had a positive impact in this area (Arabmoazzen et al., 2015).

Rodents who have had their bilateral olfactory bulbs removed have a continuously altered brain state with intricate behavioral and neurochemical alterations, many of which are similar to those experienced by people who have a severe depressive disorder (Hellweg et al., 2007). A study by Filho et al. (2016) has shown that the natural flavonoid chrysin, which is present in bee propolis, honey, and a variety of plants, has an impact that is similar to an antidepressant in chronically stressed rats. Chrysin administration is able to ameliorate the depressive-like and reverse the alteration of the elevation of tumor necrosis factor-α, interferon-γ, interleukin-1β, interleukin-6, kynurenine (KYN) levels and indoleamine-2,3-dioxygenase activity, as well as occasioned the decrease of 5-hydroxytryptamine (5-HT) and BDNF levels and increase KYN/tryptophan and 5-hydroxyindoleacetic acid/5-HT ratio in the hippocampus (Filho et al., 2016).

Zinc and magnesium are two minerals found in Talbinah that are linked to decreased depression. Other research has documented the link between depression and zinc deficiency. Patients who are very depressed have been linked to lower zinc serum levels (Roozbeh et al., 2011; Szewczyk et al., 2011). Thus, honey consumption is able to increase zinc level and produce anti-stress effect.

### 3.4. Anti-nociceptive potentials

The study by Aziz et al. (2014) discovered that the groups that received 1.2 g/kg and 2.4 g/kg of honey exhibited a substantial increase in tail flick delay time. The rats’ increased response time indicates that honey has analgesic properties at the levels used. Honey’s antinociceptive effects may be due in part to its impact on opioid receptors in the spinal cord (Aziz et al., 2014). Honey’s antioxidant capabilities (53.06 ± 0.41 mg of ascorbic acid equivalent per gram of honey) may potentially contribute to its analgesic benefits (Kishore et al., 2011). The antioxidants found in honey have been demonstrated to reduce nociceptive transmission by interacting with glutamate receptors in the central nervous system (Rosa et al., 2005). A reduction in calcitonin gene-related peptide (CGRP) and an increase in B2-gamma-aminobutyric acid (GABA_B_2) receptor expression in the spinal cord is likely to be responsible for the pain reduction (Pinto-Ribeiro et al., 2009). CGRP is a neurotransmitter involved in nociceptive transmission in the dorsal horn of the spinal cord, whereas GABA is an inhibitory neurotransmitter that suppresses transmission. It may modify the nociceptive transmission system, resulting in a reduction in pain response (DeLeo'n et al., 1994).

The study by Abd Aziz et al. (2019) perceived that prenatal stress rats were linked to enhanced nociceptive behavior alterations in oxidative stress markers, and spinal cord morphology in offspring exposed to prenatal stress. Honey administration minimized the modification of these parameters (Abd Aziz et al., 2019). Maternal stress has been linked to the development of aberrant behavior and changes in nociceptive responses in offspring, according to research (Hultman et al., 1997; Sternberg and Ridgway, 2003). The increased release of SOD-mediated oxidative stress indicators is caused in part by NMDA receptor activation, which causes neurotoxicity and cell death (Brittain et al., 2012a,b). Furthermore, oxidative stress has the ability to boost pain responses in offspring *via* upregulating NMDA receptors in the central nervous system (Betzen et al., 2009). Flavonoid treatment from honey was linked to lower nociceptive behavior scores in diabetic rats (Narenjkar et al., 2011) and neuropathic pain in rats (Azevedo et al., 2013). Pregnant dams’ dietary flavonoids can pass the placenta and be deposited in the fetal brain and other tissues (Elst et al., 1998). It is possible that the flavonoid prevented the modification of nociceptive responses. This is accomplished by altering signaling cascades and gene expression in nociceptors. As a result, serotonergic and GABAergic inhibitory neurons are protected and reduce the NMDA receptor overexpression in the rat offspring’s central nervous system (Abd Aziz et al., 2019).

One study conducted by Mohd Shafie et al. (2022) described that honey administration can reduce oxidative stress in the thalamus and was connected to pain behavior in a sleep-deprived rat model with rapid eye movement (REM). In this interesting analysis, they suggested that sleep disruption and pain have a bidirectional link. Inadequate sleep can affect pain responses and pain severity can cause sleep disturbance (Silva et al., 2004). Honey administration decreased central nervous system inflammation in rats by lowering TNF-, IL-1, glial fibrillary acidic protein, allograft inflammatory factor 1, and COX-2 levels. Honey may promote the inhibitory mechanism that reduces pain behavior score in late phase 2 of the formalin test. The finding above confirms that, honey administration was related to a reduction in pain behavior score with greater antioxidants and reduced MDA levels in the thalamus in this study. Honey is a natural product that has a high concentration of phenolics and flavonoids, both of which have anti-inflammatory and antioxidant properties (Ranneh et al., 2021). The binding of neurotransmitters to NMDA receptors modifies nociceptive pathways, resulting in enhanced pain responses (Brittain et al., 2012a,b). The antioxidants in the thalamus had a reasonable association with the pain behavior score. A positive correlation was found between the pain behavior score and the MDA level and honey administration has reduced pain behavior score and oxidative stress in the thalamus (Mohd Shafie et al., 2022).

A recent study conducted by Hasim et al. (2020b) was done to assess whether the rat offspring’s thalamus was modulated by honey administration especially histology, oxidative stress parameters, and N-methyl-D-aspartate (NMDA) receptors. In recent years, it is reported that up to 20% of pregnant women endure stress and depression (Glover and Barlow, 2014). According to reports, prenatal stress can cause pregnancy difficulties as well as disruptions in child development and behavior (Betts et al., 2014; Den Hove et al., 2014). Prenatal stress has been linked to alterations in nociceptive responses in rats and children, according to research (Davis et al., 2010). The administration of honey to pregnant dams subjected to prenatal stress produced comparable outcomes in adult rat offspring. The stress group substantially reduced the number of Nissl-positive neurons in the VPL of prenatally stressed male rats’ pups (Hasim et al., 2020b). This suggested that the antioxidants in honey, such as flavonoids, may have been transmitted to the developing fetus and resulted in long-term positive benefits (Elst et al., 1998). Furthermore, the delivery of honey to pregnant dams increased antioxidant activity and decreased oxidant levels in the rat progeny. The current study only assessed the amount of NMDA receptors in the thalamus and did not look at the different subtypes of NMDA (Hasim et al., 2020b). The NR2B subtype of the NMDA receptor is critical for nociception. As a result, future research should look at distinct NMDA receptor subtypes. This concluded that honey administration was related to decrease nociceptive behavior, as well as decreased histological alterations and oxidative stress in the thalamus (Hasim et al., 2020b).

In addition, the finding regarding albino rats that consumed honey lowered the perception of pain, particularly inflammatory pain in which the use of tamsulosin and propranolol (adrenergic blockers) spread the impact of honey (Owoyele et al., 2013). This finding revealed that the administration of tamsulosin and propranolol at the early stage of the formalin test did not affect the anti-nociceptive action of honey. However, in the last stage of the test, tamsulosin and propranolol both improved the anti-nociceptive effects of honey.

Next, a study had analysed the impact of the antinociceptive and antioxidative effects of honey and vitamin C in rats with formalin-induced pain (Hasim et al., 2020a). Previously, Aziz et al. (2014) reported that honey treatment was related to an increase in tail flick delay time when triggered with noxious heat. One of the antioxidants in honey is vitamin C, which has been proven to reduce nociceptive behavior produced by formalin injection (Rosa et al., 2005). The vitamin C group’s pain behavior score and catalase level were not statistically different from the control group. The antioxidant catalase level had a substantial and negative association with the mean pain behavior score, indicating the antioxidant’s role in influencing the pain response in the inflammatory pain model (Hasim et al., 2020a). Oxidative stress has been linked to the development of pain, such as back pain in humans (Inanır et al., 2013) and neuropathic pain in rats (Pottabathini et al., 2014). Injection of formalin causes a chain reaction of cellular processes that leads to inflammation. Inflammatory cells are drawn to the site of inflammation, and metabolism increases with increased oxygen consumption at the inflamed area. The events cause an increase in the release and buildup of ROS (Ibi et al., 2008). It is possible that it has antinociceptive properties. The effects are connected to a higher catalase level. This will give a foundation for investigating the therapeutic honey’s ability to prevent or diminish inflammatory discomfort as tabulated in Table 5.

**Table 5:** Anti-nociceptive potentials of honey supplementation on animal models.

**Authors (Year)**	**Type of source**	**Purpose**	**Mechanism**
Aziz et al. (2014)	Animal study (rat)	To examine the preemptive effects of administering different doses of honey and prednisolone on the nociceptive response in male Sprague–Dawley rats.	Honey’s antioxidant capabilities may potentially contribute to its analgesic benefits by the reduction in CGRP and an increase in (GABAB2) receptor expression in the spinal cord
Abd Aziz et al. (2019)	Animal study (rat)	To determine whether honey could prevent the altered nociceptive behavior, with its associated changes of oxidative stress markers and morphology of the spinal cord, among the offspring of prenatally stressed rats	Flavonoids from honey consumption decrease the release of SOD-mediated oxidative stress and downregulate NMDA receptors in the central nervous system.
Mohd Shafie et al. (2022)	Animal study (rat)	To investigate whether oxidative stress in the thalamus was correlated with the pain behavior score in the rapid eye movement (REM) sleep-deprived rat model.	Honey supplementation lowered oxidative stress in REMs rat models’ thalamus, modulating pain behavior in the formalin test.
Hasim et al. (2020b)	Animal study (rat)	To determine whether the modulation of nociceptive behavior by honey was mediated by modulating changes in the histology, oxidative stress parameters, and NMDA receptors in the thalamus of the rat offspring.	The delivery of honey to pregnant dams increased antioxidant activity and decreased oxidant levels in the rat progeny.
Owoyele et al. (2013)	Animal study (rat)	To investigate the analgesic and anti-inflammatory effects of honey and the effects of concurrent administration of autonomic nervous system blocking drugs.	Honey supplementation lowered the perception of pain, particularly inflammatory pain in which the use of tamsulosin and propranolol spread the impact of honey.
Hasim et al. (2020a)	Animal study (rat)	To compare the antinociceptive and antioxidative effects of honey and vitamin C in formalin-induced pain in the rat.	One of the antioxidants found in honey is vitamin C and vitamin C has been proven to reduce nociceptive behavior by increasing the antioxidant catalase level.

## 4. Conclusion

**Figure 3:**
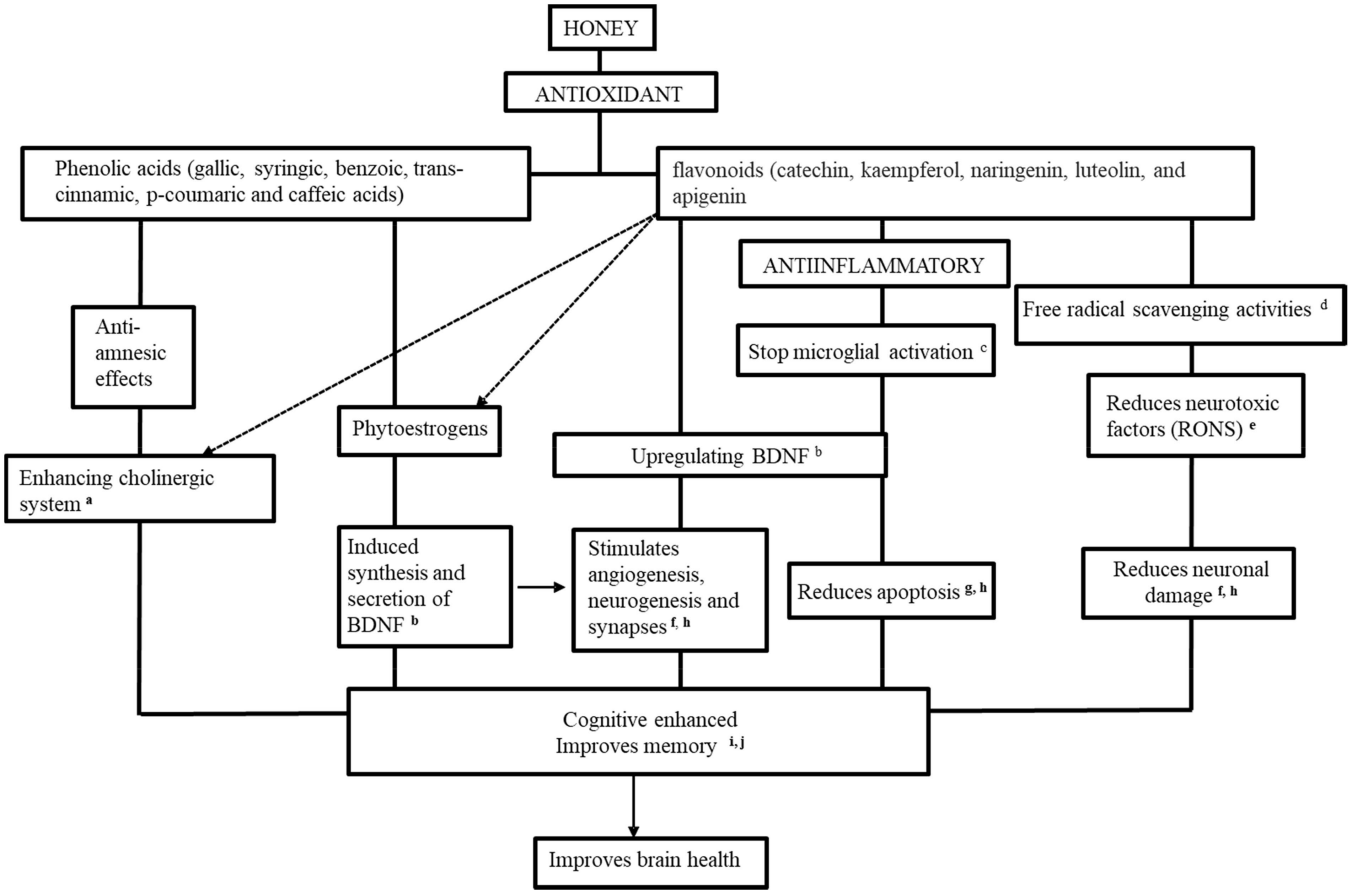
The potential therapeutic mechanisms of honey as a promising brain booster. ^a^Al-Himyari (2009), ^b^Al-Rahbi et al. (2014a), ^c^Mohd Sairazi et al. (2018), ^d^Mohd Shafie et al. (2022), ^e^Hasim et al. (2020a), ^f^Abd Aziz et al. (2020), ^g^Qaid et al. (2020a), ^h^Adeniyi et al. (2022), ^i^Yahaya et al. (2020), and ^j^Othman et al. (2011).

Our review listed the potential neurological mechanisms of honey’s involvement in memory booster, neuroprotective impact, anti-stress, and anti-nociceptive benefits which contributed to improving brain health. The presence of the phenolic content (gallic, syringic, benzoic, trans-cinnamic, p-coumaric, and caffeic acids) and flavonoids contents (catechin, kaempferol, naringenin, luteolin, and apigenin) in honey work as an antioxidant and anti-inflammatory agent to enhance cognitive and improve memory and eventually works as brain booster as Figure 3.

Most of the phenolic and flavonoid compounds mentioned above were addressed from the honeyconsumption; therefore, it assumed that the inhibition of neurotoxic factors production by the honey extracts was due to these compounds. The improvement of morphology-related brain structures, the enhancement of the cholinergic system, and inhibition of neuroinflammatory and microglial activation are due to honey supplementation.

These findings may help in the creation of novel therapeutic functions for honey, such as amyotrophic lateral sclerosis (Aaron et al., 2016; Maya et al., 2018), and Parkinson’s disease (Yildiz et al., 2013; Topal et al., 2020), as well as establishing brain nourishment (Chakraborty et al., 2013; Saxena et al., 2014; Khan et al., 2017; Mert et al., 2018; Joshi et al., 2019). More study is needed to characterize honey’s bioactive compounds, molecular processes, and key components that determine nootropic activity in order to build this new potential quality standard. Furthermore, sustainable apicultural practices should be encouraged, particularly in tropical rainforest regions.

## Author contributions

NZ and NS contributed to preparing the manuscript. NG, CI, and RZ contributed to reviewing the manuscript. All authors have read and agreed to the published version of the manuscript.

## Funding

The funding will be covered by USM.

## Conflict of interest

The authors declare that the research was conducted in the absence of any commercial or financial relationships that could be construed as a potential conflict of interest.

## Publisher’s note

All claims expressed in this article are solely those of the authors and do not necessarily represent those of their affiliated organizations, or those of the publisher, the editors and the reviewers. Any product that may be evaluated in this article, or claim that may be made by its manufacturer, is not guaranteed or endorsed by the publisher.
